# Prevalence and determinants of hysterectomy in India

**DOI:** 10.1038/s41598-023-41863-2

**Published:** 2023-09-04

**Authors:** Dejalin Rout, Abhinav Sinha, Subrata Kumar Palo, Srikanta Kanungo, Sanghamitra Pati

**Affiliations:** ICMR-Regional Medical Research Centre, Bhubaneswar, Bhubaneswar, 751023 Odisha India

**Keywords:** Health care, Risk factors

## Abstract

Increase in the prevalence of hysterectomy among low-and middle-income countries (LMICs) such as India has become a significant concern. Reports based on either a particular group or region show an increasing trend in hysterectomy, but there is a dearth of national-level data in this domain. Hence, there seems to be an urgent need to garner evidence on the prevalence and determinants of hysterectomy, which could pave the way for future programs and policies. We aimed to estimate the prevalence of hysterectomy and assess its determinants using a nationally representative sample. An observational analysis was conducted using data from Longitudinal Aging Study in India (LASI), 2017–2018. 38,154 women aged > 18 years were included. A multivariable logistic regression, presented as an adjusted odds ratio (AOR) with a 95% confidence interval (CI), was used to predict the association between various socio-demographic characteristics and hysterectomy. A separate multivariable logistic regression model was executed to determine the association between selected non-communicable diseases (NCDs) and hysterectomy. Survey weights compensated the complex study design. The overall prevalence of hysterectomy was around 11.35%. Excessive menstrual bleeding followed by fibroids emerged as the leading causes of hysterectomy. The various determinants of hysterectomy were urban residents [AOR: 1.54 (1.21–1.96)], other backward class [AOR: 2.19 (1.72–2.78], working women [AOR: 1.19(1–1.42)] and the most affluent (rich) group [AOR: 2.06 (1.62–2.63)]. Hysterectomy was associated with cancer [AOR: 4.83 (2.51–9.29)], diabetes [AOR: 1.79 (1.25–2.57)], hypertension [AOR: 1.48 (1.27–1.71)] and joint diseases [AOR: 1.43 (1.09–1.88)]. Hysterectomy is considerably prevalent in India, which cannot be overlooked. Health promotion regarding hysterectomy and its implications is needed especially among urban residents, affluent groups and those with a higher body mass index. Health programmes aimed at women should follow a life course approach by prioritizing health and overall well-being even after reproductive years.

## Introduction

Hysterectomy is the clinical procedure of removing the uterus and its surrounding structure^1^. The main reasons for undergoing hysterectomy include uterine cyst or fibroids, uterine prolapse; sliding of the uterus from its normal position into the vaginal canal; cancer of the uterus, fallopian tube, cervix, and ovaries; heavy menstrual bleeding; pain in pelvic region; and thickening of the uterus^2^. Hysterectomy can be of various types, such as: (a) supracervical or subtotal hysterectomy: removal of the upper part of the uterus but does not harm the cervix; (b) total hysterectomy: removal of the whole uterus and cervix; (c) radical hysterectomy: removal of the whole uterus, tissue on the sides of the uterus, the top part of the vagina, and the cervix,; and (d) radical hysterectomy: done in case of malignancies^1,3^.

According to the World Health Organization (WHO), globally, approximately 1,540,000 women underwent hysterectomy in the year 2016^4^. A study conducted in 2018, reported the prevalence of hysterectomy to be around 17 per 1000 ever-married women in India that varied from 2 to 63 per 1000 women across different states^5^. Further, it observed hysterectomy to be the most common major surgery performed among women^5^. Hysterectomy is mostly seen among females aged 40–45 years, and by 65 years of age, approximately 37–39% of the women undergo this surgery^4^. Social determinants such as age, age at marriage, literacy, and socioeconomic status are often associated with hysterectomy^6,7^.

Women prefer hysterectomy to prevent various health-related complications such as cancer, fibroids, uterine prolapse, and many uterine disorders^8^. Peripartum hysterectomy differs widely across different geographical regions due to differences in the anatomical structures of the uterus. It is associated with the increased phenomenon of previous caesarean delivery, placenta praevia, and morbid adherent placenta (MAP) in women^9^. However, investigating the nationwide prevalence of hysterectomy is not just based on the anatomical differences in the structure of the uterus but also on various other factors, such as an increase in the number of young women preferring to undergo hysterectomy, which has become a major concern in India^10^. Additionally, a study conducted in a low-income setting in Ahmedabad, India reported that hysterectomy was the main reason for hospitalization and insurance claims^11^. Moreover, recent studies have also raised the question over the necessity of hysterectomy as the substantial part of the hysterectomies is performed in private health facilities over public^12^.

Although focus on women’s health is concentrated mainly through the Reproductive, Maternal, Neonatal, Child and Adolescent Health (RMNCH+A) program in a life course approach; still preventing hysterectomy remains a daunting target. Recently, hysterectomy has gained attention among the health service providers as well as policymakers^13,14^. A significant increase in hysterectomy cases, along with the involvement of young premenopausal women from lower socio-economic strata, has become a grave concern^15^. Various evidence suggests easy availability of healthcare facilities due to health insurance has led to increase in hysterectomy^11^, which makes it pertinent to generate evidence on the factors determining hysterectomy in India. This would help in strengthening the existing programs and forming future policies related to healthcare of midlife women in India. Therefore, we estimated the prevalence of hysterectomy and assessed its determinants among women in India utilizing a nationally representative data from Longitudinal Aging Study in India, wave-1.

## Methods

### Overview of data

The Longitudinal Aging Study in India (LASI) wave-1, conducted during 2017–2018, was designed to examine a nationally representative sample of India’s population aged ≥ 45 years and their spouses (irrespective of age). It is a multidisciplinary panel study among ageing population in India. LASI eligible households (LEH) (with participants of the eligible age) formed the unit of observation. LASI is a partnership among the Harvard School of Public Health (HSPH), the International Institute of Population Sciences (IIPS), and the University of Southern California. LASI study was designed to capture the social and health characteristics. A multistage stratified area probability cluster sampling design was used for achieving the ultimate unit of observation. LASI, wave-1 considered a four-stage sampling design in urban areas and a three-stage sampling design in rural areas; its details can be referred from LASI, India report^16^. LASI observed a non-response rate of 12.7%.

**Figure 1:**
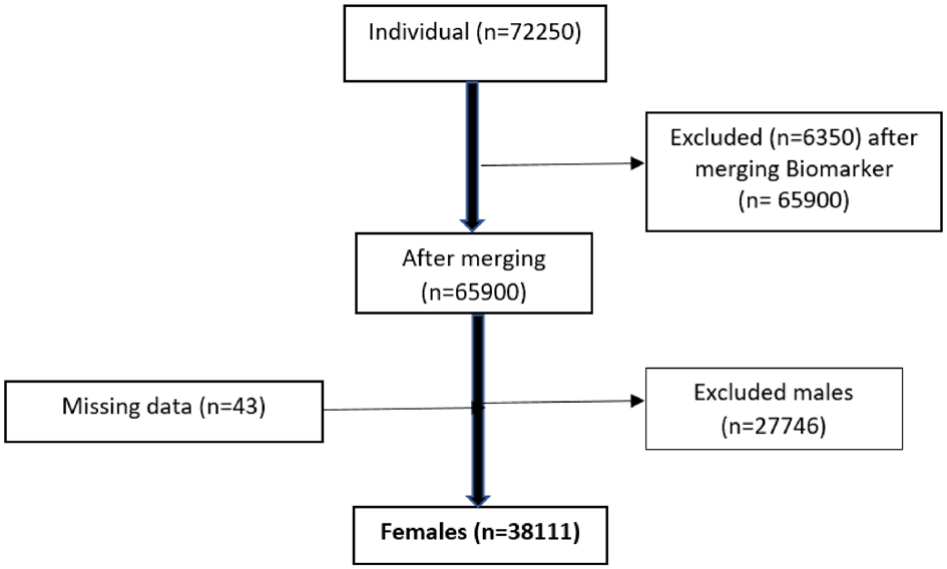
Flowchart depicting selection of study participants.

LASI included 72,250 individuals aged 45 years and above and their spouses (irrespective of their age). For our study, we merged two different datasets i.e. individual data (n = 72,250) with biomarker dataset (n = 65,900). Following our objective, 38,154 women aged > 18 years were included as our study participants. Figure 1 depicts the selection of study participants.

### Outcome variable

To assess the outcome of interest, the following question was used “have you undergone an operation to remove your uterus (hysterectomy)?” with the responses as ‘yes’ or ‘no’. The participants who responded ‘yes’ were considered as ‘underwent hysterectomy’. Further, women who responded ‘yes’, were further enquired regarding the reasons for the hysterectomy.

### Independent variable

We incorporated the following socio-demographic variables in our analysis: age in years categorized as 18–44, 45–59, ≥ 60 years; residence (urban or rural); caste (scheduled caste, scheduled tribe, other backward class, others); education (with formal education, no formal education); marital status (ever married, never married); age at first marriage (< 18 years, 19–32 years, ≥ 33 years); and number of children/parity (nulliparous: no child, uniparous: one child and multiparous: more than one child). Additionally, socioeconomic characteristics such as occupation (currently working, currently not working) and household wealth index based on monthly per capita expenditure (MPCE) classified as poor, middle and rich class. Individual characteristics such as health insurance was recorded as ‘yes’ or ‘no’. Behavioural characteristics such as physical activity was assessed on the basis of taking part in sports or vigorous activities, such as running or jogging, swimming, going to a health center or gym, cycling, or digging with a spade or shovel, heavy lifting, chopping, farm work, fast bicycling, or cycling with loads with those who responded as performing these activities everyday, more than once a week, once a week, one to three times a month being classified as ‘physically active’; and those who performed these activities hardly ever or never being termed as ‘physically inactive’. Body Mass Index (BMI) was assessed as weight (in kg) divided by height (in m^2^). Height was measured using stadiometer and weight (in kilograms) using a Seca 803 digital weighing scale. BMI was categorized as underweight (min-18.500000), normal weight (18.500000/24.99999), overweight (25/29.99999), obese (30/max)^17^.

Based on extensive literature search, the following self-reported non-communicable diseases (NCDs) were selected for the study: hypertension, cancer, high cholesterol, chronic lung disease, chronic heart disease, stroke, diabetes, arthritis, and psychological disorders. The detailed description of all variables used in analysis is presented in supplementary table S1.

### Statistical analysis

STATA version 16.0 (STATA Corp., Texas) was used for data analysis. Descriptive statistics were presented as mean with standard deviations. Frequencies and percentages were calculated for categorical variables along with 95% confidence interval (CI) for all weighted proportions as a measure of uncertainty. Binary logistic regression formed the measure of association between an exposure and the outcome, expressed as odds ratio (OR). A multivariable logistic regression, presented as adjusted odds ratio (AOR) with 95% CI, was used to predict the association between various socio-demographic characteristics and hysterectomy. A separate multivariable logistic regression model was executed to determine the association between various selected NCDs and hysterectomy. All analysis was done by utilizing survey weights to compensate for complex study design of the survey.

### Ethics approval and consent to participate

The present study utilizes de-identified data from a secondary source. The data has been archived in the public repository of LASI held at IIPS. The access to the data requires registration which is granted specifically for legitimate research purposes. LASI received mandatory ethical approval from the Indian Council of Medical Research and Institutional Review Board (IRB) held at IIPS, Mumbai. At the unit level, individuals were supplied with a catalogue containing the information on the purpose of the survey, confidentiality, and safety of health assessment. Written consent forms were administered at household and individual levels, in accordance with the Human Subject Protection. LASI data is archived in a public repository; therefore, there is no need for additional ethical approval to conduct the present study. All methods were carried out in accordance with the relevant guidelines and regulations.

## Results

### Descriptive characteristics of study population

The mean age of the study participants was 56 ± 12 years. The majority of the participants were in the age group of 45–59 years. Two third of the participants resided in rural areas. 57% of the women did not have any formal education. Nearly one fourth of the respondents had health insurance coverage. Only 3% of the women were nulliparous. Although, 72% of the participants were physically inactive still 48% had an average weight. The detailed description of participant characteristics can be seen in Table 1.

**Table 1:** Descriptive statistics of study population.

Characteristics	Category	Frequency (n)	Percentage (%)
Age in years (n = 38,154)	18–44	6057	16%
	45–59	17,237	45%
	> 60	14,824	39%
Age at marriage (in years) (n = 37,626)	< 18	24,523	65%
	19–32	12,693	34%
	33 and above	410	1%
Marital status(n = 38,153)	Ever married	37,842	99%
	Never married	311	1%
Residence (n = 38,154)	Rural	24,594	64%
	Urban	13,560	36%
Caste (n = 37,806)	Scheduled Caste	6437	17%
	Scheduled Tribe	6686	18%
	Other Backward class	14,360	38%
	Others	10,323	27%
Education (n = 38,153)	No formal education	21,729	57%
	Primary completed	8305	22%
	Up to Secondary/diploma	6939	18%
	Graduate and above	1180	3%
Occupation (n = 38,152)	Working	19,837	52%
	Not working	18,315	48%
Health insurance(n = 38,073)	Yes	8674	23%
	No	29,399	77%
No of children (n = 36,884)	Nulliparous	1031	3%
	Uniparous	2438	7%
	Multiparous	33,415	91%
MPCE quintile (n = 38,154)	Poor	15,248	40%
	Middle	7737	20%
	Rich	15,169	39%
Physical activity (n = 38,121)	Physically active	10,622	28%
	Physically inactive	27,499	72%
Body mass index (n = 37,763)	Underweight	6364	17%
	Normal weight	18,299	48%
	Overweight	9181	24%
	Obese	3919	10%

**Table 2:** Prevalence of hysterectomy across various socio-demographic characteristics of the study population.

Variable	Category	Hysterectomy n, % (95% CI)
Age (years)	15–44	665, 11.5 (10.7–12.4)
	45–59	2048, 12.3 (11.8–12.8)
	> 60	1611, 10.2 (9.7–10.6)
Age at marriage	< 18	2989, 11.1 (10.7–11.4)
	19–32	1292, 12.3 (11.6–12.9)
	33 and above	17, 8.4 (4.8–12.8)
Marital status	Married	4317, 11.4 (11.1–11.7)
	Unmarried	7, 2.2 (0.8–4.3)
Residence	Rural	2550, 9.8 (9.4–10.1)
	Urban	1774, 14.6 (14–15.3)
Caste	Scheduled cast	773, 10.4 (9.7–11.17)
	Scheduled tribe	186, 5.7 (4.9–6.5)
	Other Backward Class	2299, 13.3 (12.7–13.8)
	Other	1039, 10.5 (9.9–11.1)
Education	No formal education	2449, 10.5 (10.08–10.8)
	Primary completed	878, 11.62 (10.9–12.4)
	Up to Secondary/diploma	754, 12.45 (11.6–13.3)
	Graduate and above	243, 21.62 (19.2–24.1)
Occupation	Working	2502, 12 (11.5–12.4)
	Not working	1823, 10.5 (10–11)
Health insurance	Yes	977, 12.7 (11.9–13.4)
	No	3329, 10.9 (10.6–11.3)
No of children	Nulliparous	116, 12.1 (10.1–14.3)
	Uniparous	231, 9.5 (8.4–10.8)
	Multiparous	3870, 11.5 (11.2–11.9)
Physical activity	Exercise	1452, 13.6 (12.9–14.2)
	no exercise	2869, 10.4 (10.1–10.8)
MPCE quintile	Poor	1364, 8.4 (8–9.9)
	Middle	821, 10.5 (9.8–11.2)
	Rich	2139, 15 (14.4–15.6)
Body mass index	Underweight	442, 6.1 (5.5–6.6)
	Normal weight	1863, 10.2 (9.8–10.7)
	Overweight	1283, 15 (14.3–15.8)
	Obese	707, 18.3 (17.1–19.6)

### Prevalence and distribution of hysterectomy

The overall prevalence of hysterectomy was around 11.35%. The highest (12.3%) prevalence of hysterectomy was reported among participants aged 45–59 years. Respondents who were married between 19 and 32 years of age had higher prevalence (12.3%) of hysterectomy than other age groups (Table 2). The prevalence of hysterectomy was found to be more (11.4%) among married women. Hysterectomy was more prevalent among urban women (14.6% vs. 9.8%) than their rural counterparts. Women with formal education were observed to have higher prevalence (12.7%) of hysterectomy. We observed hysterectomy to be common among nulliparous women (12.1%). Women with a health insurance reported higher (12.7%) prevalence of hysterectomy.

### Reasons for hysterectomy

**Figure 2:**
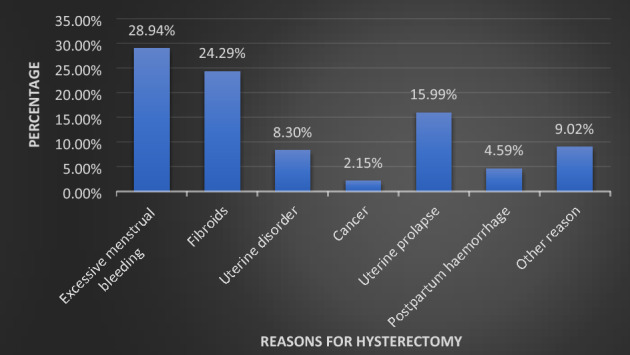
Reasons for hysterectomy in India, LASI wave-1.

We observed excessive menstrual bleeding as the leading cause for hysterectomy followed by fibroids, uterine prolapse uterine disorders, postpartum haemorrhage and cancers (Fig. 2).

### Determinants of hysterectomy

The bivariate analysis depicted hysterectomy to be associated with married, urban, other backward class, affluent class (rich) and obese women (Table 3). In the multivariable regression model, marital status was not included due to multi-collinearity. We observed women from urban areas had a higher [AOR: 1.54 (1.21–1.96)] chance of getting hysterectomy done as compared to their rural counterparts. Women belonging to other backward classes had a higher likelihood [AOR: 2.19 (1.72–2.78] of undergoing hysterectomy. Physical exercise was found to be associated [AOR: 1.43 (1.18–1.74] with hysterectomy. Women with BMI more than or equal to 25 kg/m^2^ had higher chances [AOR: 2.17 (1.68–2.82)] of undergoing hysterectomy. Affluent group had a higher likelihood [AOR: 2.06 (1.62–2.63)] of getting hysterectomy done.

**Table 3:** Association between hysterectomy and various socio-demographic characteristics.

Variable	Category	OR (95% CI)	p-value	AOR (95% CI)	p-value
Age in years	18–44	Reference		Reference	
	45–59	1.07 (0.68–1.68)	0.747	1.26 (0.95–1.70)	0.110
	≥ 60	0.86 (0.54–1.39)	0.563	1.15 (0.82–1.63)	0.405
Age at marriage (in years)	< 18	1.36 (0.73–2.501)	0.322	1.42 (0.70–2.89)	0.332
	19–32	1.52 (0.79–2.91)	0.199	1.35 (0.67–2.71)	0.394
	33 and above	Reference		Reference	
Marital status	Ever married	5.65 (1.83–17.42)	**0.003**	Omitted due to collinearity	
	Never married	Reference			
Residence	Rural	Reference		Reference	
	Urban	1.58 (1.22–2.05)	**0.000**	1.51 (1.21–1.88)	**0.000**
Caste	Scheduled caste	1.92 (1.50–2.45)	**0.000**	1.84 (1.41–2.39)	**0.000**
	Scheduled tribe	Reference		Reference	
	Other Backward Class	2.52 (1.92–3.30)	**0.000**	2.17 (1.72–2.75)	**0.000**
	Others	1.94 (1.56–2.42)	**0.000**	1.55 (1.16–2.07)	**0.003**
Education	No formal education	Reference	0.070	Reference	0.426
	Primary completed	1.12 (0.97–1.31)	0.126	0.92 (0.77–1.09)	0.359
	Up to Secondary/diploma	1.21 (0.82–1.81)	0.334	0.87 (0.59–1.28)	0.485
	Graduate and above	2.35 (0.76–7.27)	0.136	1.36 (0.55–3.38)	0.508
Occupation	Working	1.15 (0.92–1.44)	0.191	1.17 (1.01–1.37)	**0.046**
	Not Working	Reference		Reference	
Health insurance	Yes	1.18 (0.86–1.60)	0.290	1.14 (0.94–1.38)	0.178
	No	Reference		Reference	
MPCE quintile	Poor	Reference		Reference	
	Middle	1.46 (1.18–1.79)	**0.000**	1.25 (1.02–1.52)	**0.027**
	Rich	2.20 (1.64–2.96)	**0.000**	1.82 (1.51–2.19)	**0.000**
No of children	Nulliparous	1.30 (0.57–2.96)	0.517	1.69 (0.74–3.88)	0.214
	Uniparous	Reference		Reference	
	Multiparous	1.23 (0.76–2.01)	0.390	1.58 (0.95–2.62)	0.077
Physical activity	Physically active	1.34 (1.04–1.73)	**0.021**	1.42 (1.18–1.71)	**0.000**
	Physically inactive	Reference		Reference	
Body mass index	Underweight	Reference		Reference	
	Normal weight	1.76 (1.48–2.1)	**0.000**	1.55 (1.30–1.86)	**0.000**
	Overweight	2.72 (2.05–3.61)	**0.000**	2.18 (1.72–2.77)	**0.000**
	Obese	3.46 (2.2–5.44)	**0.000**	2.43 (1.77–3.32)	**0.000**

Significant values are in [bold].

### Association between selected NCDs and hysterectomy

**Figure 3:**
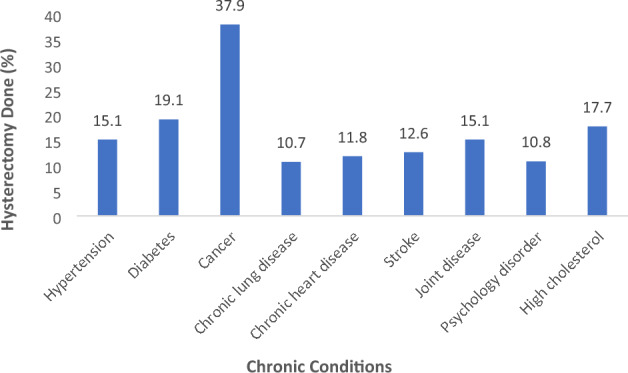
Prevalence of hysterectomy across selected NCDs.

The prevalence of hypertension was found to be around 30% followed by joint diseases (16%), diabetes (11%) and chronic lung diseases (5%) among the study participants (supplementary table S2). The prevalence of hysterectomy was highest among participants having cancer (37.9%) followed by diabetes (19.1%) high cholesterol (17.7%) and joint diseases (15.1%). The detailed description of the prevalence of hysterectomy across selected NCDs is presented in Fig. 3.

The bivariate analysis suggested hysterectomy was associated with hypertension, diabetes, cancer, joint disease and high cholesterol. The multivariable analysis suggested participants with cancer had the highest chance of undergoing hysterectomy [AOR: 4.83 (2.51–9.29)] after adjusting for age and health insurance (Table 4). Diabetes was strongly associated with hysterectomy [AOR: 1.79 (1.25–2.57)]. Participants having hypertension [AOR: 1.48 (1.27–1.71)] and joint diseases [AOR: 1.43 (1.09–1.88)] had a higher odds of undergoing hysterectomy.

**Table 4:** Logistic regression showing association between dependent variables and Non-communicable diseases.

Non-communicable diseases	Category	OR (95% CI)	p-value	AOR^a^ (95% CI)	p-value
Hypertension	Yes	1.63 (1.30–2.06)	**0.000**	1.48 (1.27–1.71)	**0.000**
	No	Reference		Reference	
Diabetes	Yes	2.03 (1.36–3.04)	**0.001**	1.79 (1.25–2.57)	**0.001**
	No	Reference		Reference	
Cancer	Yes	4.87 (2.59–9.17)	**0.000**	4.83 (2.51–9.29)	**0.000**
	No	Reference		Reference	
Chronic lung disease	Yes	0.94 (0.65–1.36)	0.753	0.85 (0.58–1.24)	0.405
	No	Reference		Reference	
Chronic heart disease	Yes	0.73 (0.46–1.16)	0.187	0.72 (0.44–1.18)	0.202
	No	Reference		Reference	
Stroke	Yes	1.05 (0.71–1.54)	0.791	0.94 (0.60–1.48)	0.812
	No	Reference		Reference	
Joint disease	Yes	1.51 (1.10–2.06)	**0.009**	1.43 (1.09–1.88)	**0.010**
	No	Reference		Reference	
Psychology disorder	Yes	0.94 (0.68–1.31)	0.743	0.81 (0.58–1.15)	0.248
	No	Reference		Reference	
High cholesterol	Yes	1.71 (1.3–2.24)	**0.000**	1.27 (0.93–1.72)	0.121
	No	Reference		Reference	

Significant values are in [bold].

^a^Adjusted for age and health insurance.

## Discussion

Gender is an important determinant of health outcomes. Women often face disparities in accessing healthcare facilities and have minimal say in the decision making for their own health especially in LMICs such as India^18^. This study estimated the prevalence of hysterectomy in India and assessed its determinants. We observed a considerable prevalence of hysterectomy. The main reasons for hysterectomy were excessive menstrual bleeding followed by fibroids. The highest prevalence of hysterectomy was found among nulliparous women. The various determinants of hysterectomy were other backward class, urban residents, working women, most affluent (rich) group, and obesity. Hysterectomy was associated with hypertension, diabetes, cancer and joint diseases.

We observed 11.3% prevalence of hysterectomy among our study population which is higher than the findings of a study utilizing the data from District Level Household and Facility Survey (DLHS-4), 2012–2013 where the prevalence of hysterectomy ranged between 0.2 and 6.3 per 100 women in the age group of 15–49 years^5^. Our findings are higher than the results of a study based on National Family Health Survey, 2015–2016 (NFHS-4) data, which reported a prevalence of 6% among women aged 30–49 years^15^. Here, it is worth noting that the prevalence of hysterectomy has almost doubled in less than a decade which could be a major concern in the future. A recent study conducted using NFHS-5 (2019–2021), estimated the prevalence of hysterectomy to be around 3.3% among women aged 15–49 years which is lower than the present study^19^. A probable reason for this could be the different age groups considered in these studies. Nonetheless, this study also revealed that the prevalence was highest (9.7%) for women aged 40–49 years^19^. A similar study conducted in China during 2017 reported the prevalence of hysterectomy to be around 7% among women aged 45–54 years which is comparatively lower than our study^20^.

The prime self-reported causes of hysterectomy were excessive menstrual bleeding/pain followed by the presence of fibroids/cysts in this study. Our findings were consistent with the findings of another study from Haryana which showed excessive menstrual bleeding (74%), uterine prolapse (10%) and fibroids (3%) were the major indications for hysterectomy^21^. However, in the United States, the main reasons reported for hysterectomy were leiomyoma (41%) followed by endometriosis (17.7%), and uterine cancer (9.2%) among women aged 15 years and above^22^. Various studies showed fibroids followed by prolapse of the uterus as the chief reason for hysterectomy^23–26^. A recent study conducted in West Bengal reported menorrhagia, leiomyoma, abnormal uterine bleeding, and pelvic organ prolapses as the major indications for hysterectomy^27^. Another study from Nagpur reported fibroid uterus (65.33%) to be the most common indication for hysterectomy^28^.

We observed other backward classes had a higher association with hysterectomy which is similar to the findings of another study based on a nationally representative data^29^. Socio-economic inequalities and access to healthcare determine the treatment offered, as conservative treatment cannot be offered to patients who seek medical care late which could be one of the probable reason for the OBCs to have higher hysterectomy. BMI was found to be a predictor of hysterectomy which is consistent with the findings of a review which suggests an increase in weight is directly proportional to an increase in the prevalence of hysterectomy^30^. Our findings are also similar to a study done in India which suggests hysterectomy to be associated with high BMI^31^. Additionally, studies document that obesity may require pre-operative evaluation for deciding abdominal or vaginal hysterectomy, may require an ad in intra-operative techniques and may also lead to postoperative complications^32,33^.

Our findings suggest urban residents had higher chances of undergoing hysterectomy than their rural counterparts. This is in contrast to the findings of a study conducted in Gujarat which reported the prevalence of hysterectomy to be higher among rural women^34^. A probable reason for this could be the regional differences as Gujarat has a high per capita income along with a well-developed healthcare infrastructure. Additionally, the hysterectomy procedure is also related to lower age at the time of first childbirth and untreated reproductive health conditions which are higher in rural areas leading to a higher prevalence of hysterectomy as found in this study^35^. Secondly, rural areas have their own beliefs, and practices regarding health, and neglected health care seeking might elevate the risk factors resulting in hysterectomy^36^. Nonetheless, a probable reason for urban residents having higher chances of hysterectomy could be an easy access to healthcare facilities, and opting for such surgeries also. Here, it is worth noting that a multi-centric study reported that the mothers in Asia had a 23% higher likelihood of hysterectomy than those in Africa which further strengthens our notion that higher and easy access to surgical facilities in Asia led to higher hysterectomies as the same study stated that Asia witnessed 37% caesarean sections as compared to only 25% in Africa^37^.

We observed affluent women were more likely to undergo hysterectomy. These findings are similar with the observations of a study which reported higher wealth status to be associated with higher odds of hysterectomy^10^. A probable reason for this could be the availability of better healthcare facilities among affluent groups due to their ability to pay. We also observed hysterectomy to be associated with the working women, which backs our notion that the increase in spending capacity of women might also increase the prevalence of hysterectomy in the future. It is pertinent to inform, educate and communicate with the working women who are also economically well-off. Nonetheless, to avoid inequity in accessing healthcare facilities, health insurance schemes such as Ayushman Bharat might be an effective means by reducing out-of-pocket expenditure and providing quality treatment to the marginalized groups^38^. Such public-funded schemes should increase their ambit to include the needs of women’s health. Nonetheless, a qualitative study conducted among rural Indian women reported hysterectomy as a secure and permanent solution to long-tern work and security as they have to bear the responsibilities of caretaker of the family^39^.

We observed hypertension to be the most prevalent chronic condition followed by joint diseases, diabetes and chronic lung diseases which is consistent with the findings of our previous study which found hypertension, gastrointestinal disorders, musculoskeletal disorders, diabetes, and obesity to be the most common chronic conditions among women in midlife^40^. In our study, women having NCDs such as obesity, hypertension, diabetes, and cancer had a higher likelihood of undergoing hysterectomy. Our findings corroborate with the findings of a cohort study among Danish population, which reports hypertension to be associated with the increased risk of hysterectomy^41^. Several studies depict obesity, diabetes, and hypertension are associated with the development of risk factors for hysterectomy^42,43^. Hypertension is also reported to increase the risk of endometrial cancer, another indication for hysterectomy^44^. The physiological mechanisms for the association between these chronic conditions and hysterectomy may be linked to decline in ovarian function and reduction in the levels of oestrogen. Nonetheless, there is a need to explore and understand the specific role if reproductive hormones in the risk of chronic diseases and hysterectomy. However, a cohort study from Taiwan showed hysterectomy to be linked as an important risk factor for various chronic conditions^45^. A recent cohort based follow-up study among North Indian women found folate repletion, and high triglyceride to be associated with hysterectomy^46^.

### Implications for policy and practice

Although traditionally, women’s health is garnered importance through programs such as RMNCH+A, still this does not continue beyond reproductive years. Expanded programs aiming life course approach beyond reproductive age may strengthen the Additionally, around midlife, women tend to develop multiple chronic conditions which may predispose to complications leading to hysterectomy, hence these NCDs and their risk factors should be targeted at an early age. Strengthening primary care can be of immense importance in providing timely, equitable and quality health services to this group. Health and Wellness Centres can be an opportunity to provide comprehensive preventive and curative care. Awareness regarding the need for hysterectomy and its effects on overall health should be the priority especially for working women and affluent group as found in this study is required.

### Strengths and limitations

This study utilized data from LASI, wave-1 thus, giving a nationally representative estimate. However, self-reported prevalence of hysterectomy and selected NCDs may have led to underestimation of the conditions due to recall bias. Several chronic conditions such as hypertension and diabetes may be undiagnosed in some cases which could undermine the true population prevalence while using self-reported data. Moreover, self-reported data leads to misclassification bias which leads to under-representation of the issue being studied^47^. Nonetheless, previous studies have also suggested a high agreement between self-reported conditions and clinically assessed conditions^48^. Moreover, the study did not report any information on the history of hysterectomy. Additionally, we could not establish temporal and potential causality as this is a cross-sectional study and longitudinal data will be required to confirm the direction of these relationships.

## Conclusion

Hysterectomy is considerably prevalent in India which cannot be overlooked. Urban, working and affluent groups were at a higher risk of hysterectomy which shows an urgent need to inform, educate and communicate the indications and effects of hysterectomy among this group. Additionally, women from deprived sections should be included in public-funded insurance schemes to eliminate disparities in seeking care. Health programmes aiming at women should follow a life course approach by prioritizing health and overall well-being even after reproductive years. Future studies should consider using data from medical records to confirm the diagnosis. Longitudinal studies are warranted to establish the causality.

### Supplementary Information

Supplementary Tables.

## Data Availability

The dataset analysed during the current study is available in the LASI data repository held at ICT, IIPS [https://iipsindia.ac.in/content/lasi-wave-i]. Requests to access the data should be made to datacenter@ipsindia.ac.in.

## Abbreviations

LMICs: Low-and middle-income countries
AOR: Adjusted odds ratio
CI: Confidence interval
NCDs: Non-communicable diseases
WHO: World Health Organization
MAP: Morbid Adherent Placenta
RMNCH+A: Reproductive, Maternal, New-born, Child and Adolescent Health
LASI: Longitudinal Aging Study in India
HSPH: Harvard School of Public Health
IIPS: International Institute of Population Science
USC: University of Southern California
BMI: Body mass index
OR: Odds ratio
ICMR: Indian Council of Medical Research
PRH: Peripartum hysterectomy
PPH: Postpartum haemorrhage
DLHS: District Level Household and facility Survey
NFHS: National Family Health Survey
IRB: Institutional Review Board
