# Supplementary material for: Prevalence and determinants of hysterectomy in India
Source: Sci Rep. 2023 Sep 4;13:14569. doi: 10.1038/s41598-023-41863-2 (PMC10477345; doi:10.1038/s41598-023-41863-2)
Supplement: Supplementary file 1 — Supplementary Tables. [file 41598_2023_41863_MOESM1_ESM.docx]

| Supplementary Table S1: Description of variables in the study, Longitudinal Ageing Study in India (LASI), 2017-18 | | |
| --- | --- | --- |
| Variables | Question | Categories |
| Age | How old were you at your last birthday? (Age in complete years) | Recoded into:  1. 18-44  2. 45–59  3. >60 |
| Age at first marriage | How old were you when you (first) got married? (Completed Age in Years) | Recoded into:   1. <18 2. 19-32 3. 33 and above |
| Marital Status | What is your current (latest) marital status? | Recoded into:  1. Ever married (currently married/widowed/divorced/separated/deserted/live-in relationship)  2. Never married. |
| Residence | Where have you lived most of your adult life? | 1. Urban 2. Rural |
| Caste | What is your caste or tribe?  Do you belong to a scheduled caste, a scheduled tribe, other backward class, or none of these? | Recoded into:  1. Scheduled caste  2. Scheduled tribe  3. Other backward class (OBC)  4. Others (no caste/tribe + none of them) |
| Education | Have you ever attended school? | Recoded into:   1. With formal education (Yes) 2. No formal education (No) |
| Occupation | Have you ever worked for at least 3 months during your lifetime? | 1. Working (Yes) 2. Not working (No) |
| Health insurance | Are you covered by health insurance? | 1. Yes 2. No |
| No of children | Number of live births. | 1. Nulliparous: no child 2. Uniparous: one child 3. Multiparous: more than one child |
| Physical activity | How often do you take part in sports or vigorous activities, such as running or jogging, swimming, going to a health center or gym, cycling, or digging with a spade or shovel, heavy lifting, chopping, farm work, fast bicycling, cycling with loads: everyday, more than once a week, once a week, one to three times a month, or hardly ever or never? | 1. Physically active (everyday/more than once a week/ once a week/ one to three times a month) 2. Physically inactive (hardly ever or never) |
| MPCE quintiles | Based on monthly per capita expenditure (MPCE) | Recoded into:   1. Poor (poorest + poorer) 2. Middle (middle) 3. Rich (richer + richest) |
| Body Mass Index (BMI) | Height (in centimetre) was measured using stadiometer and for weight (in kilograms) a Seca 803 digital weighing scale was used. BMI was enumerated as weight (in kg) divided by height (in m^2^) | Recoded as per the WHO guidelines:  1. Underweight (min-18.500000)  2.Normal weight (18.500000/24.99999)  3.Overweight (25/29.99999)  4.Obese (30/max) |
| Non-communicable Diseases | Has any health professional ever diagnosed you with the following chronic conditions or diseases? | Hypertension  Diabetes  Cancer  Chronic lung disease  Chronic heart disease  Stroke  Arthritis  Psychological disorder  High cholesterol |
| Reason for hysterectomy | What were the reason(s) for undergoing hysterectomy? | Excessive menstrual bleeding/Pain  Fibroids/cysts  Uterine disorders (Rupture)/ Injury  Cancer  Uterine prolapse  Severe Postpartum hemorrhage |

| Supplementary Table S2: Weighted prevalence of selected non-communicable diseases | | |
| --- | --- | --- |
| Non-Communicable Diseases | Frequency (n) | Percentage (%) |
| Hypertension (n=38144) | 11311 | 30% |
| Diabetes (n=38145) | 4290 | 11% |
| Cancer (n=38145) | 273 | 1% |
| Chronic lung disease (n=38146) | 1848 | 5% |
| Chronic heart diseases(n=38146) | 1072 | 3% |
| Stroke(n=38145) | 424 | 1% |
| Joint disease (n=38147) | 6175 | 16% |
| Psychiatric problems (n=38142) | 746 | 2% |
| High cholesterol (n=38144) | 1344 | 4% |
